# Genome-wide identification of actin-depolymerizing factor gene family and their expression patterns under various abiotic stresses in soybean (*Glycine max*)

**DOI:** 10.3389/fpls.2023.1236175

**Published:** 2023-07-27

**Authors:** Yongwang Sun, Deying Wang, Mengmeng Shi, Yujie Gong, Shuwen Yin, Yexuan Jiao, Shangjing Guo

**Affiliations:** School of Agricultural Science and Engineering, Liaocheng University, Liaocheng, China

**Keywords:** actin-depolymerizing factor, soybean, abiotic stresses, genome-wide identification, expression analysis

## Abstract

The actin-depolymerizing factor (ADF) encoded by a family of genes is highly conserved among eukaryotes and plays critical roles in the various processes of plant growth, development, and stress responses via the remodeling of the architecture of the actin cytoskeleton. However, the ADF family and the encoded proteins in soybean (Glycine max) have not yet been systematically investigated. In this study, 18 *GmADF* genes (*GmADF1 – GmADF18*) were identified in the soybean genome and were mapped to 14 different chromosomes. Phylogenetic analysis classified them into four groups, which was confirmed by their structure and the distribution of conserved motifs in the encoded proteins. Additionally, 29 paralogous gene pairs were identified in the *GmADF* family, and analysis of their Ka/Ks ratios indicated their purity-based selection during the evolutionary expansion of the soybean genome. The analysis of the expression profiles based on the RNA-seq and qRT-PCR data indicated that *GmADFs* were diversely expressed in different organs and tissues, with most of them responding actively to drought- and salt-induced stresses, suggesting the critical roles played by them in various biological processes. Overall, our study shows that *GmADF* genes may play a crucial role in response to various abiotic stresses in soybean, and the highly inducible candidate genes could be used for further functional studies and molecular breeding in soybean.

## Introduction

The actin cytoskeleton which is ubiquitous to all eukaryotic cells, not only provides mechanical support for the maintenance of the basic cellular structure, but is also involved in diverse biological processes that are necessary for plant growth, development, and response to various environmental changes (Porter and Day, 2016; Melak et al., 2017; Schaks et al., 2019). The organization and dynamics of the actin cytoskeleton are specific and directly regulated by numerous actin-binding proteins (ABPs) (Pollard, 2016). The actin-depolymerizing factors (ADFs) are an important class of low-molecular-mass (15 – 22 kDa) ABPs that play essential roles in many actin-remodeling processes. Their structure is characterized by the presence of a conserved motif known as the actin-depolymerizing factor homology (ADF-H) domain (Inada, 2017). The classic form of ADF can cleave or depolymerize filamentous actin (F-actin) into either shorter fragments or globular actin (G-actin), which provides new sites for the initiation of novel actin filaments and supplies more actin monomers for polymerization. Thus it regulates the reorganization and rearrangement of the actin cytoskeleton which in turn facilitates the responses to various developmental and environmental stimuli (Wioland et al., 2017).

The ADF protein was first isolated from the brains of chick embryos (Bamburg et al., 1980), and since then the *ADF* genes have been identified in a wide range of eukaryotes (Gunning et al., 2015). The organisms such as simple eukaryotes and those of animal lineages typically possess a maximum of three isoforms of ADF, whereas higher plants possess an enlarged *ADF* family (Roy-Zokan et al., 2015). The genome-wide identification of the *ADF* family has been accomplished in several plant species. For instance, the genomes of *Arabidopsis thaliana* (Feng et al., 2006; Ruzicka et al., 2007), rice (*Oryza sativa*; Feng et al., 2006; Huang et al., 2012), and tomato (*Solanum lycopersicum*; Khatun et al., 2016) each contained 11 *ADF*s. In addition, 9, 10, 13, 14, 25, and 27 *ADF*s were identified from the genomes of common bean (*Phaseolus vulgaris*; Ortega-Ortega et al., 2020), pigeon pea (*Cajanus cajan*; Cao et al., 2021), maize (*Zea Mays*; Huang et al., 2020), poplar (*Populus trichocarpa*; Roy-Zokan et al., 2015), wheat (*Triticum aestivum*; Xu et al., 2021), and banana (*Musa acuminata*; Nan et al., 2017), respectively. The expansion of gene number may allow plant *ADF* genes expressed in a complicated profile and differentiated into multiple biological functions.

Based on their phylogenetic relationship, the *ADF*s in Arabidopsis have been categorized into four groups (I – IV), with each group exhibiting a unique expression profile (Feng et al., 2006; Ruzicka et al., 2007). The members of group I (*AtADF*s *1*, *2*, *3*, and *4*) were expressed at a relatively higher level throughout the plant except in the pollen. Genes of group II were specifically expressed in the polar cells: *AtADF7* and *AtADF10* in the mature pollen grains; whereas those of *AtADF8* and *AtADF11* was limited to the root-trichoblasts and root hair. The genes of Group III (*AtADF5* and *AtADF9*) were weakly expressed in the vegetative tissues, but strongly in the rapidly growing and/or differentiating cells. *AtADF6*, a group IV gene was constitutively expressed at moderate or lower levels in all the organs and tissues (Dong et al., 2001; Ruzicka et al., 2007). This diversity in the tissue-specific expression patterns of plant *ADF*s indicated their evolution into divergent physiological functions. Indeed, biochemical experiments indicated that the ADFs belonging to groups I, II, and IV possessed a conserved F-actin cleaving/depolymerizing (D-type) activity, while the members of group III demonstrated an F-actin bundle-formation (B-type) activity, this variation resulted from certain crucial amino acid substitutions, suggesting that functional divergence had occurred in the ADF family of plants (Nan et al., 2017).

*ADF*s in higher plants have been proven to be involved in numerous biological processes, including the growth and expansion of organs, flowering, stomatal movement, and responses to various stresses (Burgos-Rivera et al., 2008; Huang et al., 2012; Fu et al., 2014; Sengupta et al., 2019). *AtADF1* is involved in regulating hypocotyl elongation and responses to salt-induced stress (Zhao et al., 2015; Wang et al., 2021). The actin dynamics mediated by *AtADF2* are essential for the infection of *Arabidopsis* roots by the root-knot nematode (Clement et al., 2009). *AtADF3* and *AtADF4* play vital roles in regulating hypocotyl growth, the morphology of epidermal cells, tolerance to drought- and osmotic-induced stress, and attacks by various pathogens (Porter et al., 2012; Henty-Ridilla et al., 2014; Zhao et al., 2016; Inada, 2017; Yao et al., 2022). In response to ABA- and drought-induced stresses, the expression of *AtADF5* was induced, which then participated in regulating stomatal closure (Qian et al., 2019). *ADF*s in crop plants have proven to be essential for the responses and tolerance to various stress. The expression of *TaADF* (Genebank No. U58278) was much higher in the freezing-tolerant wheat cultivars when compared with that in the sensitive ones (Ouellet et al., 2001). *TaADF3* and *TaADF4* demonstrated contrasting effects on the resistance of wheat plants to infection by *Puccinia striiformis* f. sp. tritici (Tang et al., 2016; Zhang et al., 2017). Overexpression of *OsADF3* and *TaADF16* improved the drought- and freezing-tolerance of transgenic *Arabidopsis* seedlings, respectively (Huang et al., 2012; Xu et al., 2021). GmADF2 interacted with the P3 protein of soybean mosaic virus (SMV), which may facilitate its infection process (Lu et al., 2015). Downregulation of *GhADF6* enhanced the abundance of actin filaments and bundles in the root cells, and rendered cotton plants tolerant to infection by the fungal pathogen, *Verticillium dahliae* (Sun et al., 2021).

Soybean is an important food and cash crop cultivated worldwide, which serves as an excellent source of plant-based proteins and edible oils which are rich in various beneficial nutrients, such as isoflavones and vitamins. Adverse environmental stresses, such as drought, salt, and extreme temperatures, greatly hindered the growth of soybean plants and thus severely diminished the yield (Deshmukh et al., 2014). To the best of our knowledge, there has been only one report regarding the soybean *ADF* gene to date (Lu et al., 2015). As the *ADF* family is known to be critical for the growth, development, and stress tolerance of plants, investigation of the family in soybean is of great importance. In this study, 18 soybean *ADF*s were identified from the genome of the soybean cultivar “Williams 82”. Next, the structures of these genes were analyzed, their locations in the genome were determined, the duplication events and phylogenetic relationships were identified, and the conserved motifs within the sequences of the encoded proteins were recognized. Tissue-specific expression profiles of these genes and in response to heat, cold, drought, and salt-induced stresses were also analyzed to determine their putative functions. The data provided in this study are reliable to screen key candidate genes from the *ADF* family in soybean for further functional investigation at molecular level, and for the molecular breeding of soybean with stress tolerance.

## Materials and methods

### Identification of the *ADF* genes in the genome of soybean

The whole genome, coding, and protein sequences of soybean (Wm82.a4.v1) were obtained from the Phytozome v13 database (https://phytozome-next.jgi.doe.gov/). The hidden Markov model (HMM) profile of the ADF-H domain (PF00657) was obtained from the Pfam database (http://pfam.xfam.org/) and was used to search for the ADF protein using the HMMER software (http://hmmer.org/). The NCBI-CDD web server (https://www.ncbi.nlm.nih.gov/cdd/) was accessed to confirm the occurrence of the ADF-H domain in the putative ADF protein. The physicochemical characteristics of the GmADF proteins, including their molecular weights (MW), isoelectric points (pI), instability index (InI), aliphatic index (AI), and grand average of hydropathy index (GRAVY) were predicted using the protParam tool of the ExPASy server (https://web.expasy.org/protparam/). Subcellular location was predicted using the WoLF PSORT tool (https://wolfpsort.hgc.jp/).

### Localization to hromosomes, gene duplication, and estimation of the Ka/Ks values of *GmADFs*

*GmADFs* were mapped to the respective soybean chromosomes based on their physical location. The duplication of these genes in the soybean genome was analyzed and visualized using the TBtools software (Chen et al., 2020). To further analyze the evolutionary divergence of the duplicated genes from each other, the nonsynonymous substitution rate (Ka) and synonymous substitution rate (Ks) of each pair of duplicated *GmADFs* were calculated using the Ka/Ks_Calculator 2.0 (Wang et al., 2010). The duplication time was calculated according to published method by using the following formula: Time = Ks/(2 × substitution rate) and the substitution rates of soybean is 6.1 × 10^-9^ site per year (Fan et al., 2013).

### Construction of the phylogenetic tree of the ADF proteins

Multiple sequence alignment-based analysis of the ADF proteins was performed using the graphical tool ClustalX 2.0 (http://www.clustal.org/). The software MEGA 11.0 (https://www.megasoftware.net/) was used to construct an unrooted phylogenetic tree using the neighbor-joining method and the bootstrap analysis was conducted using 1000 replicates.

### Analysis of the gene structure and identification of the conserved motif in the protein

The GFF3 format file of the soybean gene sequences extracted from the Phytozome v13 database was used to analyze the structures of the *GmADFs*. The exon-intron distribution chart was generated using the TBtools software. The conserved motifs of the GmADF proteins were identified using the MEME suite (http://meme-suite.org/) using the following parameters: a maximum of six motifs and optimal motif lengths of 6 – 100 amino acids.

### Tissue-specific expression patterns of *GmADF*s

The expression levels of the *GmADFs* in the roots, root hair, nodules, stems, leaves, shoot apical meristem (SAM), pods, and seeds were analyzed based on the soybean-RNA-Seq datasets obtained from the Phytozome v13 database. The expression levels were summarized as Reads Per Kilobase of Transcript per Million mapped reads (RPKM), and a heatmap indicating the tissue-specific expression profiles was generated using the log2-transformed (RPKM + 1) values of the GmADFs which were analyzed with the TBtools software.

### Plant growth and stress treatments of soybean seedlings

The seeds of “Williams 82” were allowed to germinate on absorbent paper for three days and then transferred to half-strength Hoagland solution and grown under a 16-h-light/8-h-dark photoperiod and a 25°C/18°C (day/night) cycle. The plants were exposed to the stress-inducing conditions of heat (37°C), cold (4°C), salt (150 mM NaCl), and drought (20% w/v PEG-6000) for 0, 1, 3, 6, 12, and 24 h after the emergence and uncurling of the first leaf. Thereafter, the roots were collected, flash-frozen in liquid N_2_ and then stored at −80°C until the subsequent step of RNA extraction. The samples were collected from five individual plants exposed to the same treatment, for every type of treatment, which was done in triplicates.

### qRT-PCR analysis

The total RNA of the samples were extracted using a RNA plant extraction kit (TSINGKE, cat. TSP401, China) according to the manufacturer’s instruction. Briefly, roots were ground into fine powder in liquid N_2_, and then transfer 100 mg powder to 0.45 µl Buffer RL. The RNA was purified using the RNase-Free Columns CS and finally eluted in 30 µl of RNase-free ddH_2_O. The NanoDrop 2000c spectrophotometer (Thermo Scientific) was used to measure the quantity and quality of RNA. The RNA concentration of all samples was in the range of 300 - 500 ng/µl, and the 260/280 and 260/230 ratios were all around 2.0. One microgram of RNA was reverse-transcribed to generated the first-strand cDNA was generated using a Prime Script RT reagent kit (TSINGKE, cat. TSK301S, China) and stored at –20°C until use. Quantitative real-time polymerase chain reaction (qRT-PCR) was performed on a LightCycler^®^ 480 system (Roche, Basel, Switzerland) using SYBR Green qPCR kits (Cat. No. Q223, Vazyme, Nanjing, China). The PCR program used consisted of 95°C for 300 s followed by 40 cycles at 94°C for 10 s, 60°C for 10 s and 72°C for 15 s. Relative quantification of gene expression was performed based on the 2^−ΔCt^ method (Derveaux et al., 2010) using *GmTub* (*Glyma.05G157300*) as the internal control following a published protocol (Wang et al., 2016). The data was analyzed and expressed as graphs using GraphPad prism 8.0 (https://www.graphpad.com/). The values mentioned are mean averages of the measurements made in triplicates. The statistical significance of these was assessed by Tukey’s pairwise comparison test. The sequences of the gene-specific primers used in this study are listed in **Supplementary Table** **S1**.

## Results

### Identification of *ADF* Genes in soybean

**Figure 1:**
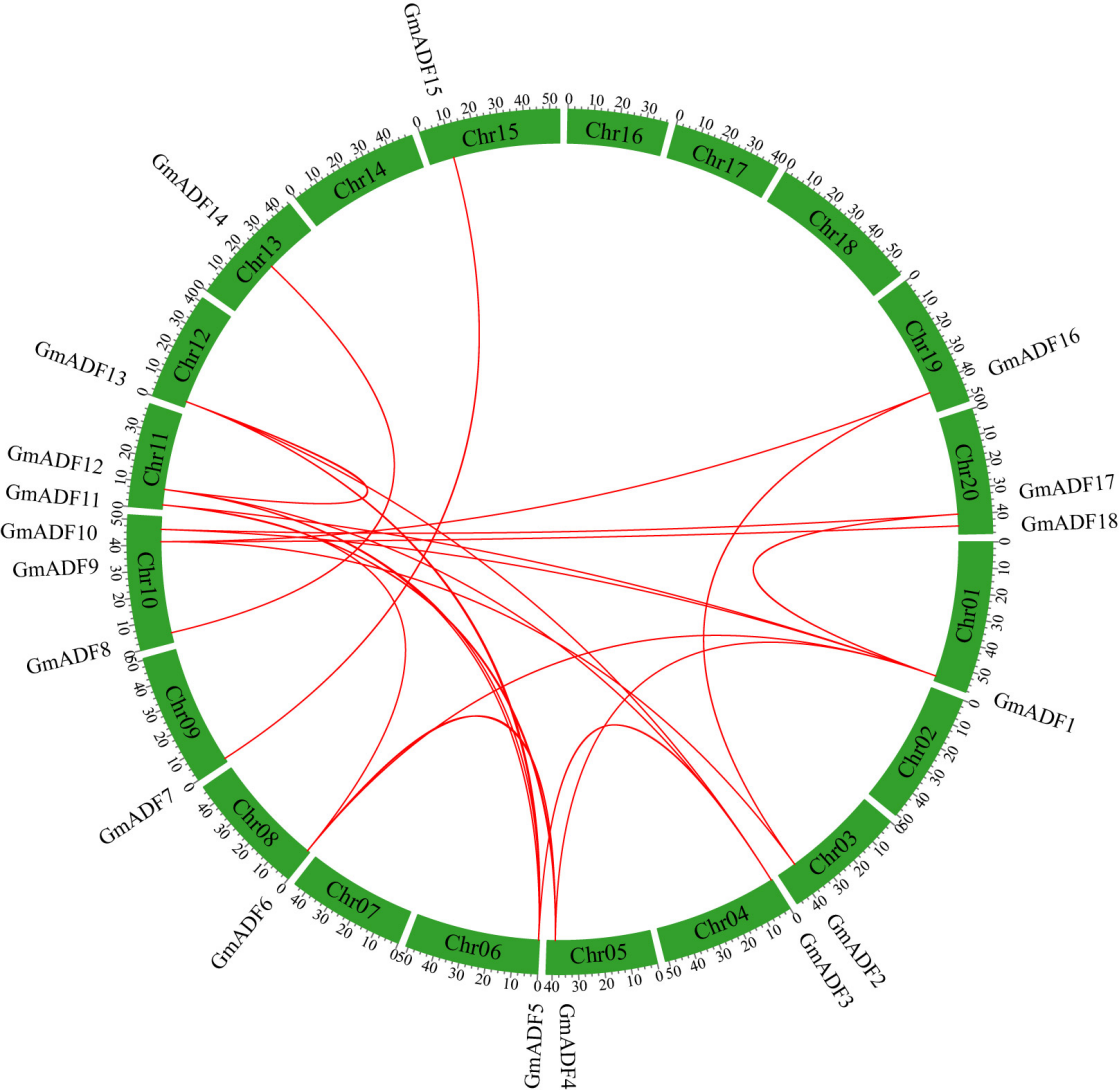
Distribution and synteny analysis of *GmADF* genes on the chromosomes of the soybean genome. The locations of the *ADF*s on the chromosomes are shown on the outside. The colored boxes indicate the different chromosomes (Chr1 – Chr20). The colored lines connecting the *GmADF*s located on the different chromosomes represent segmental duplication events.

A total of 18 putative *ADFs* were identified in the soybean genome and were designated as *GmADF1 – GmADF18* based on their chromosomal locations (**Table 1**, **Figure 1**). The GmADF proteins were generally shorter with lengths varying from 137 (GmADFs 1, 4, 6, 10, 11, and 17) to 148 (GmADFs 9 and 18) amino acids, and MWs in the range of 15.74 – 16.97 kDa. Their predicted pIs varied from 5.13 to 7.77, indicating that some ADFs tend to be acidic or basic. The GRAVY values obtained were < 0, suggesting that they possess hydrophilic characteristics. The InI values of 10 proteins > 40 indicated that they may be more unstable than the other 8 which are probably more stable with InI values ranging from 26.49 to 39.45. The AI values of 61.44 – 88.61, indicated their higher thermal stability (**Table 1**). These proteins were mainly localized to the chloroplast, followed by the cytoplasm, mitochondria, extracellular and nuclear, which indicated their functional roles in these organelles (**Supplementary Figure S1**).

**Table 1:** Detailed information on the *GmADF* genes.

Name	ID	Protein Length	MW (kDa)	pI	InI	AI	GRAVY
*GmADF1*	*Glyma.01G218900*	137	15.92	5.41	36.78	66.93	-0.469
*GmADF2*	*Glyma.03G162900*	146	16.90	7.77	41.49	61.44	-0.671
*GmADF3*	*Glyma.04G004250*	143	16.32	5.84	29.79	70.98	-0.276
*GmADF4*	*Glyma.05G206500*	137	15.80	5.49	42.98	64.09	-0.430
*GmADF5*	*Glyma.06G003900*	143	16.32	5.84	29.79	70.98	-0.276
*GmADF6*	*Glyma.08G013400*	137	15.84	5.49	44.39	64.74	-0.431
*GmADF7*	*Glyma.09G019200*	139	16.01	6.15	47.82	72.30	-0.502
*GmADF8*	*Glyma.10G044000*	139	15.98	5.92	48.19	71.65	-0.475
*GmADF9*	*Glyma.10G180700*	148	16.81	6.84	41.88	69.19	-0.467
*GmADF10*	*Glyma.10G235500*	137	15.74	5.13	39.45	74.82	-0.327
*GmADF11*	*Glyma.11G024500*	137	15.83	5.21	38.26	64.82	-0.480
*GmADF12*	*Glyma.11G106600*	143	16.30	7.65	26.49	66.85	-0.297
*GmADF13*	*Glyma.12G031700*	143	16.26	7.65	26.49	67.55	-0.279
*GmADF14*	*Glyma.13G131700*	139	16.00	5.91	47.57	71.65	-0.465
*GmADF15*	*Glyma.15G125300*	139	15.98	6.15	47.82	73.02	-0.484
*GmADF16*	*Glyma.19G164400*	146	16.97	6.91	41.15	62.12	-0.696
*GmADF17*	*Glyma.20G158900*	137	15.74	5.13	39.45	74.82	-0.327
*GmADF18*	*Glyma.20G209800*	148	16.84	6.84	40.74	69.19	-0.485

### Chromosomal location and gene duplication of *GmADF* genes

*GmADF*s were unevenly distributed on 14 out of the 20 chromosomes of the soybean genome, with 1 – 3 *GmADF*s on each (**Figure 1**). In detail, three *GmADF*s were mapped to chromosome 10, two *GmADF*s each to 11 and 20, and only one each to chromosomes 1, 3, 4, 5, 6, 8, 9, 12, 13, 15, and 19. Segmental and tandem duplication are two critical events that lead to an increase in the number of members of a gene family (Cannon et al., 2004). To further investigate the gene duplication events within the *GmADF* family, a colinear analysis was performed using the TBtools software. In total, 29 pairs of genes produced through segmental duplication were identified, but none were produced through tandem duplication (**Figure 1**). The Ka/Ks ratios of the 29 duplicated pairs were < 0.5 (**Supplementary T** **able S2**), suggesting that they have been subjected to a potentially strong selective pressure during evolution. Furthermore, it was estimated that the duplication events between *GmADF* genes might occur at 2.95 to 187.16 million years ago (MYA) (**Supplementary T** **able S2**).

### Phylogenetic relationships of the *GmADF*s

**Figure 2:**
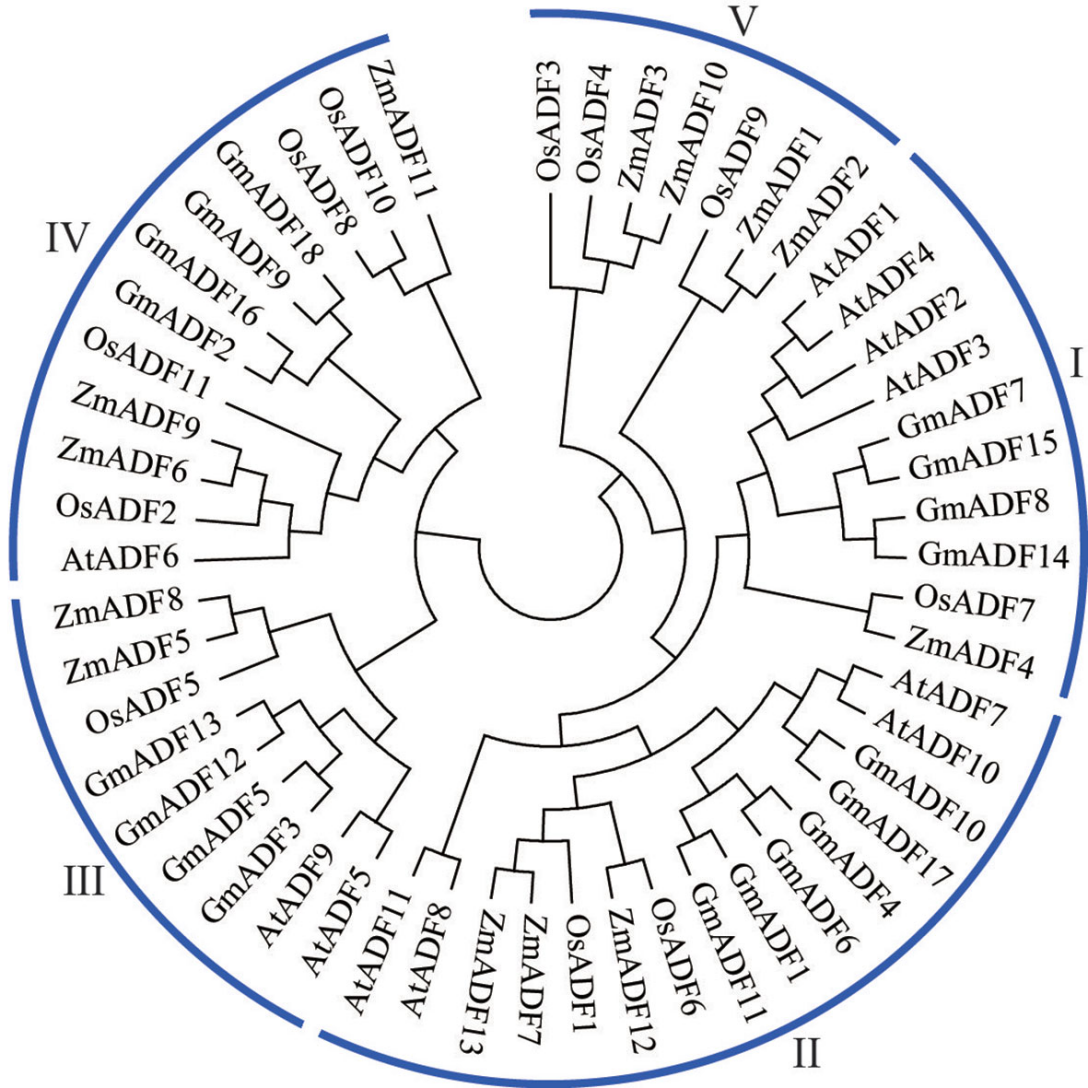
Phylogenetic analysis of the ADF proteins from soybean, *Arabidopsis*, maize, and rice. The neighbor-joining method using 1000 bootstrap replicates and the MEGA11.0 software was adopted to construct the phylogenetic tree.

To elucidate the evolutionary relationship among the *GmADF*s, a total of 53 ADF proteins, including 11 from *Arabidopsis*, 11 from rice, 13 from maize, and 18 from soybean, were used to construct a phylogenetic tree using the neighbor-joining method. As shown in **Figure 2**, the ADFs were divided into five groups with disproportional representation. In addition to group V which comprised only seven monocot ADFs, the other four groups harbored ADFs from all the four plant species. Group II consisted of the largest number of ADF proteins (15), followed by groups IV (12), I (10), and III (9). Six GmADFs clustered to group II, and four each to groups I, III, and IV.

### Structural characters of the *GmADF* genes

**Figure 3:**
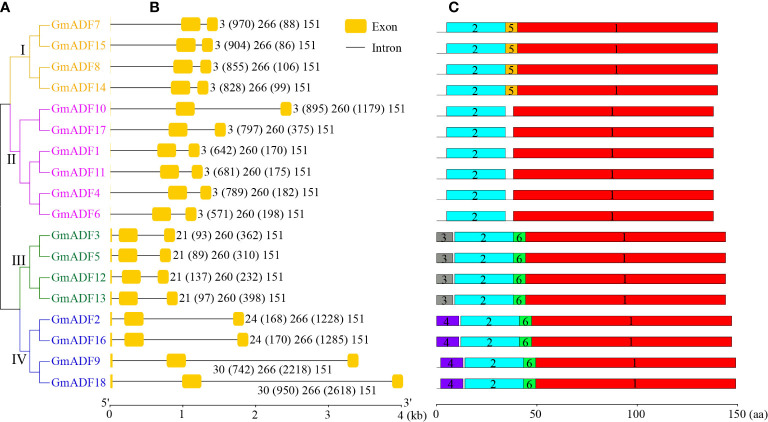
Identification of the structure of the *GmADF*s and conserved motifs of the encoded proteins based on their phylogenetic relationships. **(A)** A rootless neighbor-joining tree was constructed based on the complete sequences of the 53 ADF proteins identified from Arabidopsis, rice, maize, and soybean using the MEGA11.0 software. **(B)** The structural analyses of the exon-intron boundaries of the *GmADF*s with the yellow boxes and the black lines indicating the exons and introns, respectively. **(C)** The distribution map of the conserved motifs of the GmADFs. The six putative motifs are represented by the different colored boxes. The lengths of the exon-intron junctions and amino acid sequences were inferred by the ruler at the bottom.

The structures of the *GmADF*s were further analyzed and the results obtained demonstrated that the genes belonging to the same clade of the phylogenetic tree shared similar exon/intron structures (**Figures 3A, B**). All the *GmADF*s consisted of two introns, a 151-bp exon at the 3’-terminus, and a second exon 260-bp (groups II and III) or 266-bp (groups I and IV) long (**Figures 3A, B**). The genes of groups I and II had an extremely short first exon “ATG”, while those of groups III and IV had a longer first exon (21/24/30 bp). A comparison of the exon sites revealed that the conserved splice-sites (GT) after the ‘ATG’ codon were altered in the genes of groups III and IV, which led to splicing events occurring at the adjacent splice-sites (GT). The differences in the genomic length among the various *GmADF*s were mainly attributable to variations in the length of the introns: ten genes of groups I and II, and the *GmADF9/18* of group IV had a relatively longer first intron, whereas the second introns of the four genes from group IV and the *GmADF10* from group II were longer than those of the other 13 genes (**Figures 3A, B**).

### Characteristics of the GmADF protein sequences

The protein sequence identity among the GmADFs was > 55.30% (between GmADF4 and GmADF3/5), while the protein sequences of two pairs– GmADF3/5 and GmADF10/17 were the same (**Supplementary T** **able S3**). Multiple protein sequence alignment revealed that the ADF-H domain and the calmodulin- and the actin-binding regions were present in all the GmADFs; most of the proteins (except those from group III) contained a conserved Ser residue that might be a putative phosphorylation site (**Supplementary F** **igure S2**). Among the six conserved motifs identified in the GmADFs, Motif-1, and Motif-2 were the main regions that make up the structure of the ADF-H domain (**Figure 3C**; **Supplementary F** **igure S3**). Motif-3, Motif-4, and Motif-5 were specific to the members of groups III, IV, and I, respectively, and Motif-6 was present in GmADFs from groups III and IV (**Figures 3A, C**; **Supplementary Figure S3**).

In Arabidopsis, the ADFs from group III have evolved to demonstrate the B-type function but not the classic D-type activity, while the ADFs from group I demonstrated a stronger D-type activity than those of groups II and IV (Nan et al., 2017). Several amino acids were confirmed to be crucial for the occurrence of functional divergence among the AtADFs. Alignment of multiple GmADF sequences suggested that the specific amino acids – F5, K6, and W13 which may be critical for the B-type function of AtADF5 (Nan et al., 2017), were also present in the group III GmADFs (**Supplementary Figure S2**), suggesting that they may have a similar functional evolution. H11 was specific to group I AtADFs and critical for its enhanced D-type activity. Interestingly, both the group I GmADFs and the GmADFs 1, 11, 4, and 6 from group II harbored the H11 site (**Supplementary Figure S2**), suggesting that these proteins may have enhanced D-type activity.

### Organ- and tissue-specific expression profiles of *GmADF* genes

**Figure 4:**
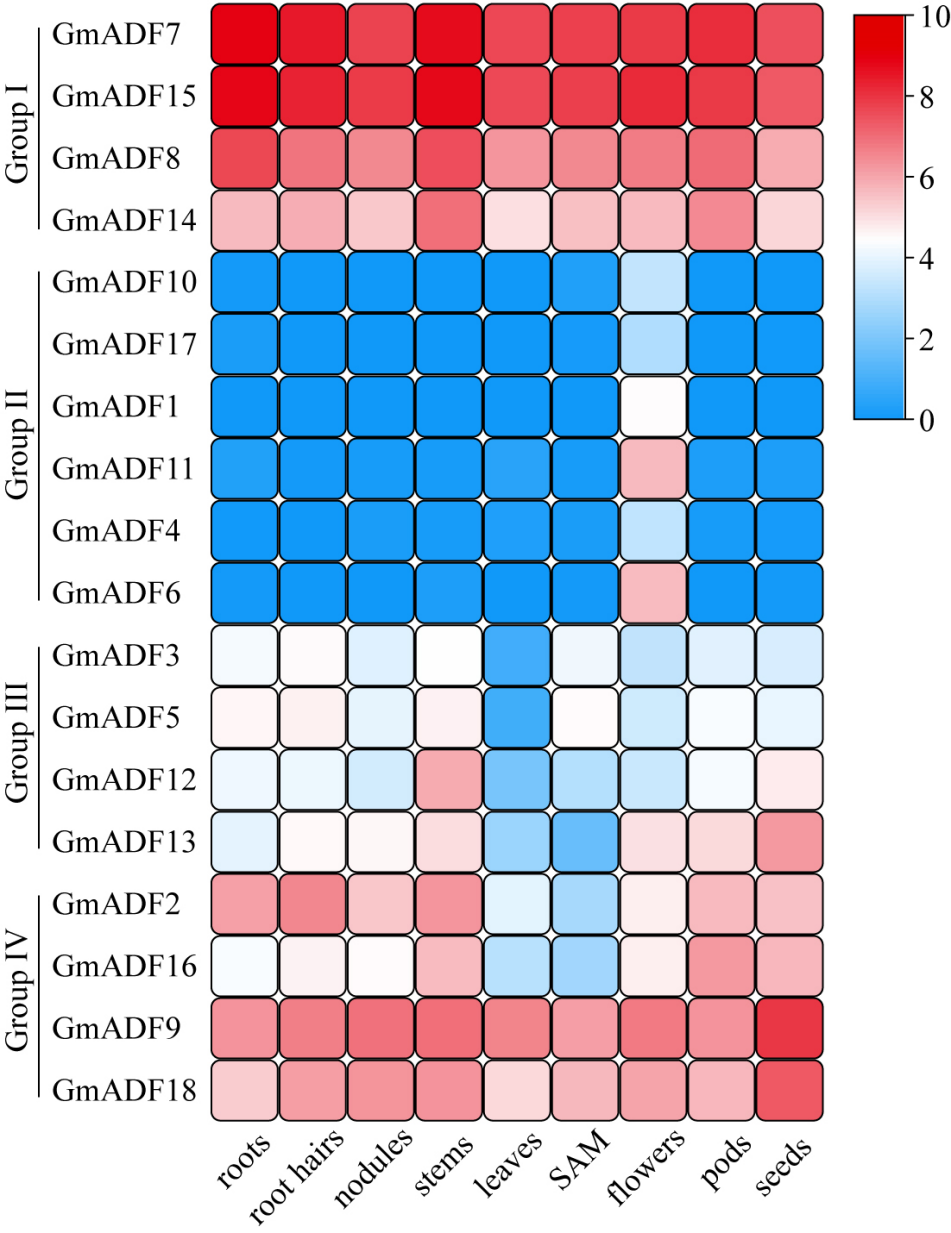
The expression profiles of *GmADF* genes. The transcription levels of the *GmADF*s in nine selected tissues or organs of soybean plants were analyzed based on the data collected from Phytozome. A heatmap was generated using TBtools. The color scale from blue to red indicates the increased expression levels of the genes.

To gain insights into the putative roles of *GmADFs*, their expression profiles in nine organs or tissues were analyzed based on the respective RNA-Seq data collected from the Phytozome database (**Figure 4**; **Supplementary T** **able S4**). In general, the GmADFs of the same group shared similar expression patterns. Eight GmADFs of groups I and IV were expressed throughout the entire plant, among which six (GmADFs 7, 8, 9, 14, 15, and 18) were expressed at a relatively higher level than the remaining two, suggesting that they play crucial roles in growth and development in soybean (**Figure 4**; **Supplementary T** **able S4**). GmADFs 2 and 16 were expressed at low levels in the leaves and the SAM, implying a pattern of tissue-specific expression. In comparison with the genes from the other three groups, those of group II showed a flower-specific expression which was suggestive of their potential roles in reproduction in soybean. Four GmADFs from group III were expressed at relatively lower levels in most of the organs/tissues (**Figure 4**; **Supplementary Table** **S4**), implying that these genes may participate in specific biological processes.

### Transcription patterns of *GmADF* genes in roots under different abiotic stresses

**Figure 5:**
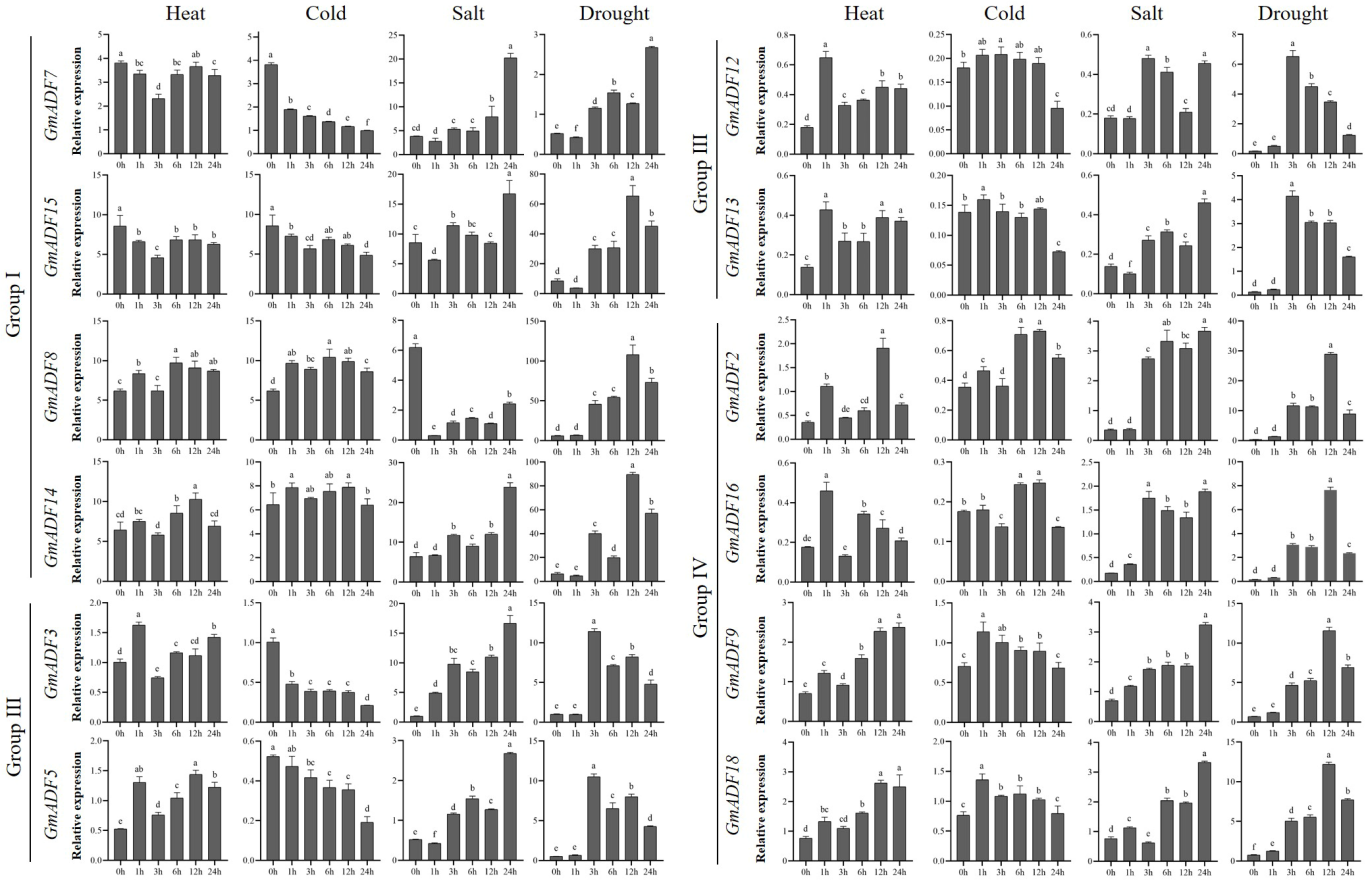
The relative expression levels of GmADFs under heat, cold, salt, and drought-induced stresses in soybean. qRT-PCR was used to study the expression levels of 12 *GmADFs* in triplicates. The abscissa indicates the time points after the stress treatments. The vertical bars indicate the standard errors of the means. The lower-case letter(s) above the vertical bars indicate statistically significant differences (P < 0.01; Tukey’s) between the various time points.

Root is the main organ for plants to absorb water and mineral nutrients, and also the primary site for sensing and coping with various abiotic stresses. To understand the putative functions of GmADFs in response to various abiotic stresses, roots of soybean seedlings exposed to conditions of heat, cold, salt, and drought were harvested and the relative expression levels of 12 root-expressed GmADFs were examined (**Figure 5**; **Supplementary Figure S4**). An alteration of > two-fold in the transcriptional levels of a gene in any of the treated samples relative to that of the control and P < 0.01 was considered to be differentially expressed. The qRT-PCR revealed that GmADFs 2 and 5 were responsive to all four abiotic stresses, indicating that they may essential for enhancing the tolerance of soybean plants to such stresses. The expression levels of GmADF2 were significantly upregulated under all four stresses; those of GmADF5 were significantly enhanced by heat, salt, and drought-induced stress, while it was repressed by cold-induced stress. Under heat-induced stress, the expression of GmADFs 2, 5, 9, 12, 13, 16, and 18 were significantly upregulated, while those of the other five genes were insensitive. Under conditions of cold-induced stress, GmADFs 3, 5, and 7 were down-regulated, the relative expression of GmADF2 after 12 h was 2.06-fold higher, while the expression of the other eight genes did not change significantly. The expression of GmADF8 was drastically downregulated by > 20-fold, whereas those of the other 11 genes showed varying degrees of upregulation under the stress induced by salt treatment. After 3 h of drought treatment, the expression levels of all 12 root-expressed GmADFs were significantly enhanced with five of them (GmADFs 2, 5, 12, 13, and 16) showing upregulation of > 20-fold compared with the control (**Figure 5**; **Supplementary Figure S4**).

## Discussion

The ADFs are among the most important ABPs with a proven involvement in various biological processes including stress tolerance in plants (Inada, 2017; Sun et al., 2021; Xu et al., 2021). Genome-wide identification and functional characterization of the ADF genes have been achieved in several plant species (Ruzicka et al., 2007; Khatun et al., 2016; Huang et al., 2020; Xu et al., 2021). However, our understanding of the ADF family in soybean is greatly limited. Hence, these genes in soybean were identified along with an analysis of their phylogeny, duplication relationship, sequences of the genes and their encoded proteins, and expression profiles. A total of 18 ADFs were identified in the soybean genome (**Table 1** **,** **Figure 1**), which is the highest among all the diploid plants analyzed to date (Feng et al., 2006; Ruzicka et al., 2007; Khatun et al., 2016; Huang et al., 2020). It has been noted that this phenomenon of the existence of a high number also occurs in other soybean gene families, such as the growth regulating factor (Chen et al., 2019) and B-box gene families (Shan et al., 2022), and may have mainly resulted from two whole genome duplication events that had occurred during the evolution of soybean (Schmutz et al., 2010). Tandem and segmental duplications are considered to be the major driving forces behind the multiplication of gene families during evolution (Cannon et al., 2004). In Arabidopsis, two duplicate gene pairs (AtADFs 1 and 2; and AtADFs 3 and 4) are arranged in tandem (Nan et al., 2017), while one tandem duplication event (TaADFs 17 and 18) had been identified in wheat (Xu et al., 2021). In contrast, only segmental duplication events were identified within ADFs from tomato (Khatun et al., 2016) and maize (Huang et al., 2020). In this study, all 29 pairs of duplicated GmADFs were identified to be generated by segmental duplication, suggesting that it was primarily responsible for the expansion of the ADF family in soybean during evolution (**Figure 1**; **Supplementary Table** **S2**). Further analysis of the evolutionary selective pressure showed that GmADFs underwent a strong purifying selection during evolution, suggesting that their functions might be evolutionarily conserved (**Supplementary** **Table S3**).

Previous studies have shown that the ADFs in flowering plants had most likely evolved from a common ancestor (Nan et al., 2017). Based on their phylogenetic relationship, 53 ADF proteins from Arabidopsis, rice, maize, and soybean were clustered into five groups (**Figure 2**); this was in agreement with the structure of their genes and the distribution of motifs within them (**Figure 3**) and also the published data (Ruzicka et al., 2007; Huang et al., 2012; Khatun et al., 2016; Huang et al., 2020). It was observed that the number of genes in the subfamilies varied among the different plant spp. For example, the distribution of ADFs to the groups I, II, III, IV, and V in Arabidopsis were 4, 4, 2, 1, and 0 respectively; which were 4, 6, 4, 4, and 0 in soybean; 1, 2, 1, 4, and 3 in rice; and 1, 3, 2, 3, and 4 in maize (**Figure 2**). These results suggested that although the ADF families in different species might have had a common ancestor, the subsequent evolutionary processes were relatively independent of each other. Correspondingly, the gene sequences and their expression patterns were also reasonably varied as described next.

Simple eukaryotes and organisms of animal lineages possess only a few ADF isoforms which have been reported to be highly conserved both structurally and functionally (Gunning et al., 2015). However, the functional divergence occurred in the higher plants as the number of individual gene members expanded to a dozen or dozens (Inada, 2017). The biochemical analysis of the ADFs from Arabidopsis showed that the group III ADFs displayed a B-type but not the classic D-type activity, and that the D-type activity of group I ADFs was stronger than those of groups II and IV (Nan et al., 2017). Multiple sequence alignment revealed that group III GmADFs had the same amino acid substitution as was identified in Arabidopsis (**Supplementary Figure S2**), suggesting that the evolution of the B-type activity of ADFs may also have occurred in soybean. It is worth noting that not only the GmADFs of group I, but also the GmADFs 1, 4, 6, and 11 of group II harbored the H11 substitution, which is critical for the enhanced D-type activity of group I AtADFs (**Supplementary Figure S2**), implying that functional differentiation through evolution may have occurred among the group II genes. Phosphorylation of a Ser at the N-terminus is critical for the modulation of the biochemical activity of ADFs in various organisms and is linked to the Ca-signaling pathway; thereby it reorganizes the cytoskeleton in response to environmental and developmental signals (Blanchoin et al., 2000; Porter et al., 2012; Dong and Hong, 2013). Multiple sequence alignment demonstrated the occurrence of an S10T substitution in the group III GmADFs, which may influence the roles played by them in biological processes such as signal transduction. These observations may indicate the existence of a diverse functional divergence within the GmADF family, which needs further investigation.

The expression patterns of genes in various organs or tissues may reflect to a certain extent the specific functional differences of the members of that gene family. The actin genes in Arabidopsis were grouped into the vegetative and reproductive classes due to their tissue-specific pattern (McKinney and Meagher, 1998). Similarly, previous studies have mentioned that the genes of the ADF family can also be divided into two classes that differ in their expression patterns, i.e., reproductive or constitutive/vegetative (Ruzicka et al., 2007). In this study, the group II GmADFs were found to specifically express in the flowers (**Figure 4**, **Supplementary** **Table S4**), which was in agreement with the published data concerning *Arabidopsis*, rice, maize, and wheat (Ruzicka et al., 2007; Huang et al., 2012; Khatun et al., 2016; Huang et al., 2020). Thus, it can be speculated that the GmADFs 1, 4, 6, 10, 11, and 17 may be critical for reproduction in soybean. The AtADFs of Group I have been proven to be highly expressed in all the tissues except in pollen and play important roles in diverse biological processes including organ growth and responses to various stresses (Clement et al., 2009; Wang et al., 2021; Yao et al., 2022). Similarly, in this study the expression levels of *GmADFs 7, 8, 14*, and *15* of group I and *GmADFs 9* and *18* of group II were higher than those of the other *GmADFs* (**Figure 4**, **Supplementary T** **able S4**), implying their essential roles in soybean development.

The rearrangement of the actin cytoskeleton of plant cells can be a target for the signaling pathways induced by numerous developmental and environmental stimuli (Melak et al., 2017; Schaks et al., 2019). Previous studies have shown that the ADFs of plants actively respond to various biotic and abiotic stresses and participate in developing resistance to them, via the remodeling of the actin cytoskeleton (Inada, 2017). Previous studies concerning the genome-wide characterization of ADFs in plants have suggested that their expression levels were significantly altered under abiotic stresses such as cold, drought, high salt, abscisic acid, jasmonic acid, wounding, etc (Khatun et al., 2016; Huang et al., 2020; Xu et al., 2021). Certain ADFs had been confirmed to possess the potential to improve stress resistance in crop plants. Overexpression of *TaADF16* and *OsADF3* enhanced the tolerance to freezing- and drought-induced stresses in transgenic Arabidopsis plants (Huang et al., 2012; Xu et al., 2021). *SaADF2* from *Spartina alterniflora*, a model halophyte for monocotyledonous grass crops, significantly enhanced the tolerance to drought- and salinity-induced stress when expressed in rice plants to a greater extent than its rice homolog *OsADF2* (Sengupta et al., 2019). Similarly, *DaADF3* isolated from *Deschampsia antarctica*, a plant native to Antarctica, played an important role in the enhancement of cold tolerance when expressed in rice plants (Byun et al., 2021). In this study, the expression levels of *GmADFs* changed to varying degrees under heat, cold, drought, and salt-induced stresses. For example, the expression levels of *GmADFs* 2, 5, 12, 13, and 16 were upregulated by > 20-fold under drought treatment (**Figure 5**). Considering the critical roles of ADFs in modulating stress tolerance in plants, the exploration of the *GmADFs* regarding their functions is needed for the elucidation of the molecular mechanisms underlying stress tolerance in soybean.

## Conclusion

In this study, a total of 18 ADF genes were identified in the soybean genome, which was unevenly distributed on 14 chromosomes. Segmental duplication during evolution was primarily responsible for the expansion of the soybean ADF family. Based on their phylogenetic relationships, these genes could be divided into four groups with those of each group showing similar structures and motif distribution in the encoded proteins. Several amino acids that are critical for the divergence of their biochemical activities were identified in the GmADFs, and biochemical analyses were needed in the future to clarify their functional properties. GmADF genes exhibited diverse expression patterns in the various tissues or organs of soybean plants, and many of them were found to respond to varied abiotic stresses, especially those induced by drought and salt, implying that they may crucial for generating stress tolerance in soybean. These genes are important candidates for the investigation of the molecular mechanisms concerning stress resistance of soybean, and also for the breeding of new soybean varieties with enhanced stress tolerance, which may enlighten soybean genetic improvement in resistance to abiotic stresses.

## Data availability statement

The original contributions presented in the study are included in the article/**Supplementary Material**. Further inquiries can be directed to the corresponding authors.

## Author contributions

YS and SG designed this study. YS and DW performed the bioinformatics analysis and the most experimental work. DW, MS, YG, SY, and YJ grew the plants and performed the stress treatment. YS and DW analyzed the data and wrote this manuscript. All authors have read and approved the final manuscript.
