# Supplementary material for: Genome-wide identification of actin-depolymerizing factor gene family and their expression patterns under various abiotic stresses in soybean (Glycine max)
Source: Front Plant Sci. 2023 Jul 27;14:1236175. doi: 10.3389/fpls.2023.1236175 (PMC10413265; doi:10.3389/fpls.2023.1236175)
Supplement: Supplementary file 1 [file DataSheet_1.pdf]

Supplementary Material

Genome-Wide Identification of Actin-Depolymerizing Factor Family Genes and Their Expression Patterns under Various Abiotic Stresses in Soybean

Yongwang Sun*, †, Deying Wang †, Mengmeng Shi, Yujie Gong, Shuwen Yin, Yexuan Jiao and Shangjing Guo*

School of Agricultural Science and Engineering, Liaocheng University, Liaocheng, China

*** Correspondence:**

Yongwang Sun

sunyongwang@lcu.edu.cn

Shangjing Guo

guoshangjing@lcu.edu.cn

† These authors have contributed equally to this work.

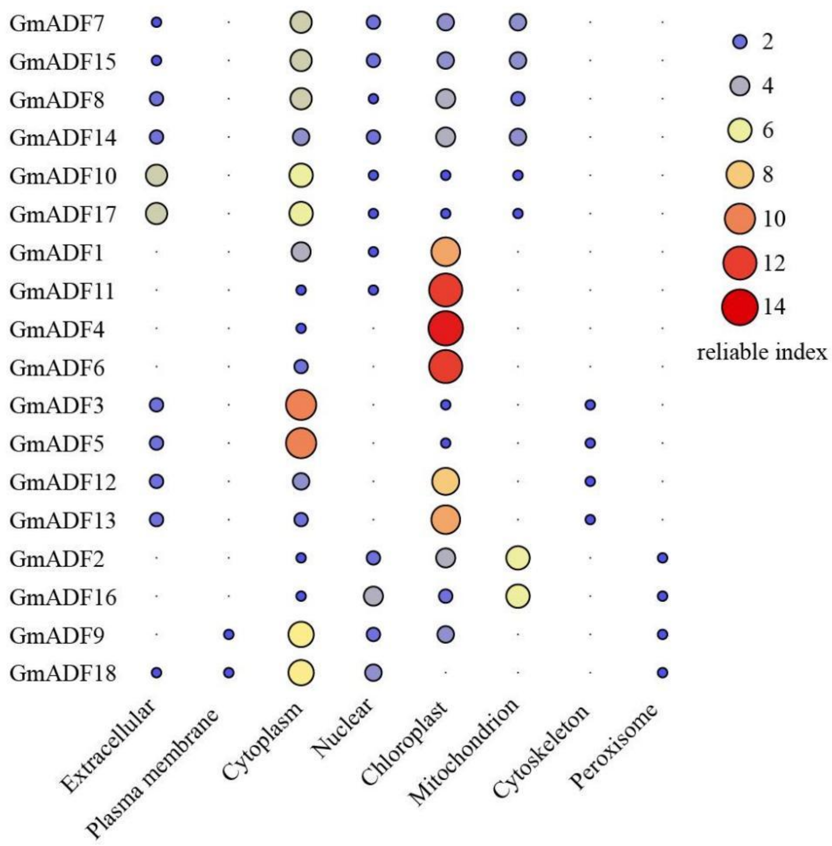

FIGURE S1 The prediction of the subcellular locations of the GmADF proteins.

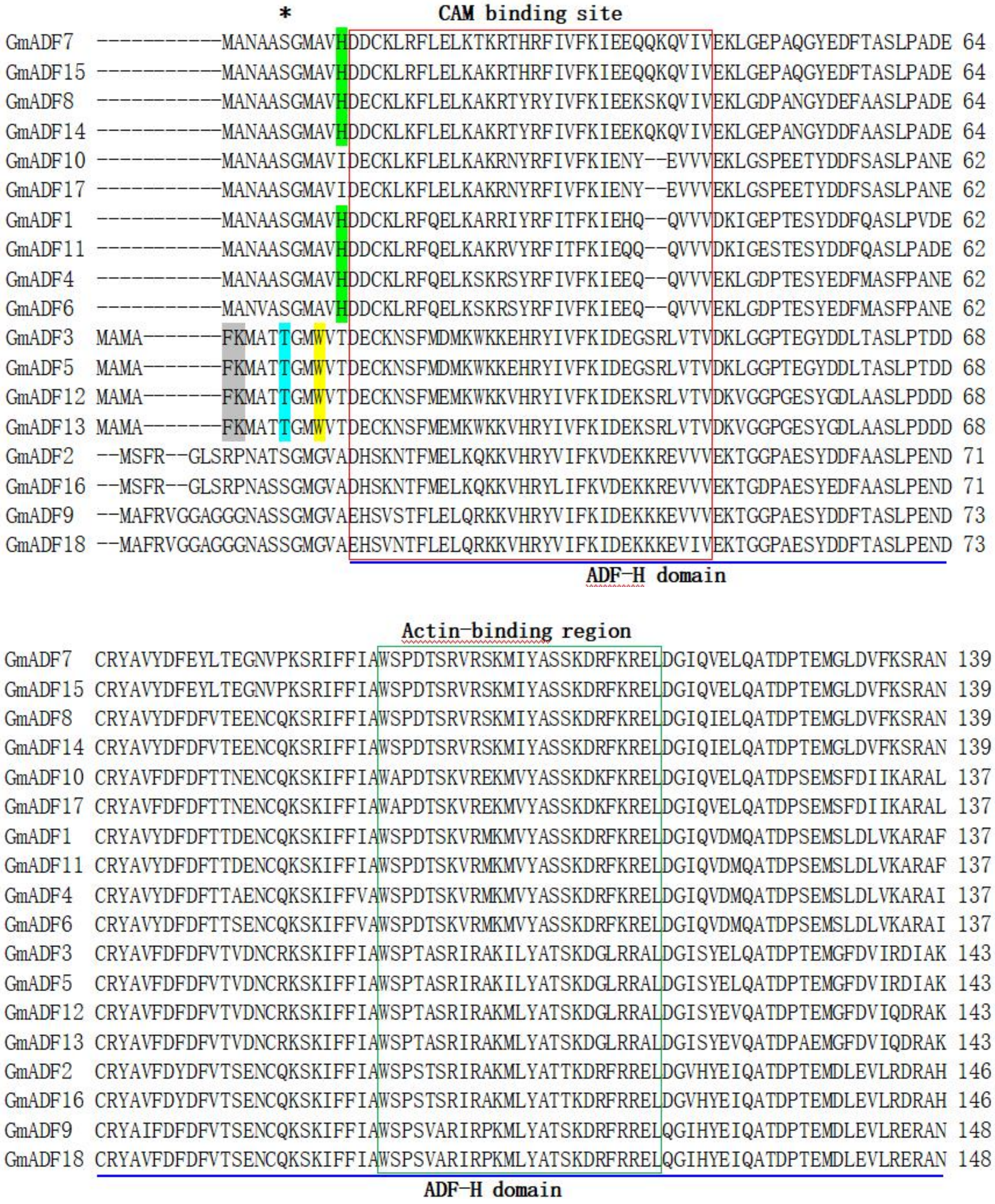

FIGURE S2 The alignment of all the deduced sequences of the ADF proteins from soybean. The asterisk indicates the putative Ser phosphorylation site. The red box indicates the putative CAM binding site and the green box indicates the amino acids essential for actin binding. The blue underline indicates the position of the ADF-H domain. The grey, cyan, green, and yellow backgrounds highlight the amino acids that are crucial for the biochemical activity of the GmADF proteins.

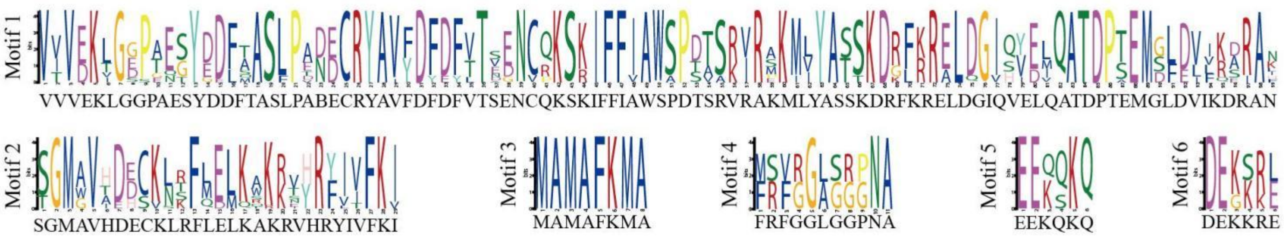

FIGURE S3 The sequences of the six conserved motifs.

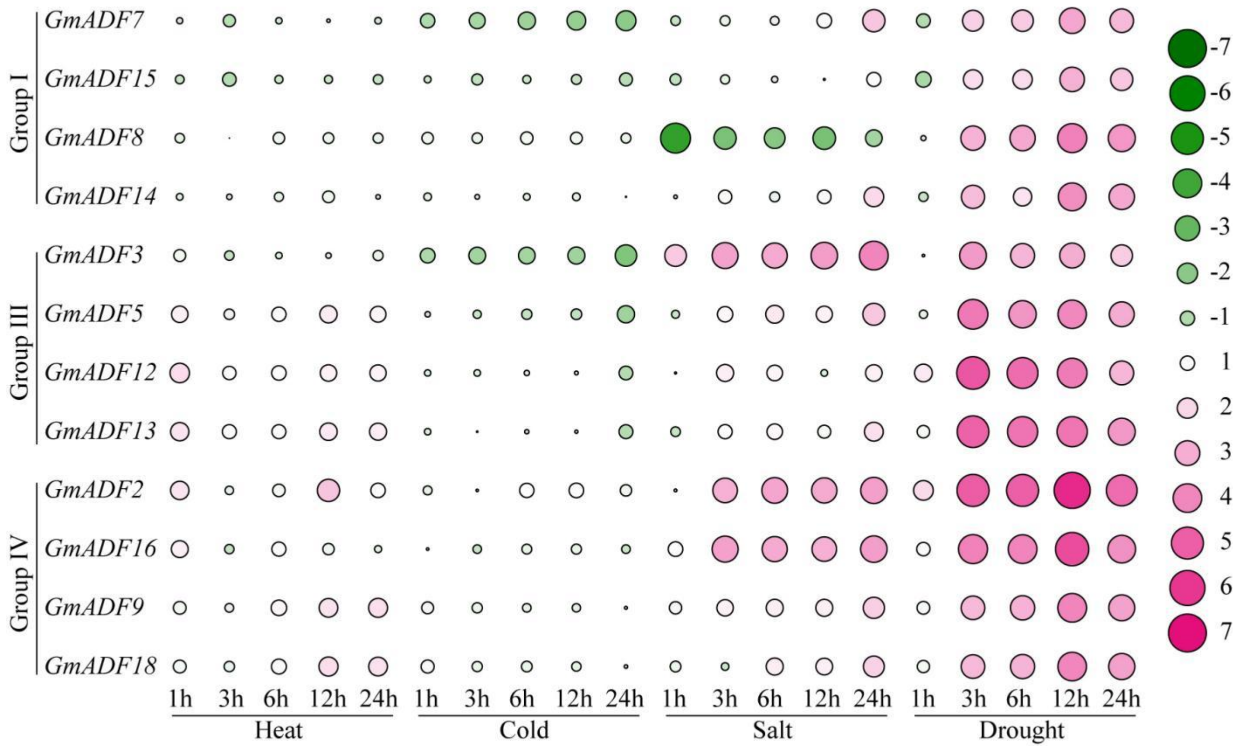

FIGURE S4. Expression profiles of *GmADF* genes under heat, cold, salt and drought stresses relative to control. Expression level of each gene at different timepoint was normalized to fold change relative to that in 0h, and the heatmap indicating the expression profiles was generated using the log2-transformed fold change.

TABLE S1 The sequences of the primers used for the qRT-PCR analysis of the *GmADF* genes

Gene name	Forward primer	Reverse primer
Tubulin	TCTTGGACAACGAAGCCATCT	TGGTGAGGGACGAAATGATCT
GmADF2	TCTCTGTCGTCGCTCTTTCT	TCTCAACCACAACCTCCCTT
GmADF3	TGGCGATGGCTTTCAAGATG	GCTCTTGCGGCAGTTATCAA
GmADF5	TGGCGATGGCTTTCAAGATG	GCTCTTGCGGCAGTTATCAA
GmADF7	AGACAAAGAGGACACACCGT	TTGCTCCTCACCCTTGATGT
GmADF8	ATTGTGGAGAAGCTGGGTGA	TCCATCAAGCTCCCTCTTGAA
GmADF9	CACTGCATCCTTGCCTGAAA	TGGGTCTGTTGCCTGAATCT
GmADF12	CGACTACTGGGATGTGGGTT	CTTGCTCTTGCGGCAGTTAT
GmADF13	TCACCGTTGATAAGGTGGGT	CCTTGGATGTTGCGTAGAGC
GmADF14	ATTGTGGAGAAGCTGGGTGA	TCCCATCAAGCTCCCTCTTG
GmADF15	GATTGAGGAGCAGCAGAAGC	TTCCATCCAGCTCCCTCTTG
GmADF16	GCAGAAGAAGGTTCACCGTTA	CGGATTCGAGACGTTGAAGG
GmADF18	CACTGCATCCTTGCCTGAAA	TGGGTCTGTTGCCTGAATCT

TABLE S2 The Ka/Ks ratios of the pairs of the duplicated *GmADF* genes

Duplicated gene pairs	Ka	Ks	Ka/Ks	Time (Mya)
GmADF7/GmADF15	0.003069058	0.119536699	0.025674609	9.8
GmADF8/GmADF14	0.024885642	0.069682579	0.357128607	5.71
GmADF1/GmADF4	0.061079476	0.374432495	0.163125468	30.69
GmADF1/GmADF6	0.067749275	0.435517682	0.155560331	35.7
GmADF1/GmADF10	0.134747913	1.544116691	0.087265369	126.57
GmADF1/GmADF11	0.015504428	0.100358247	0.154490823	8.23
GmADF1/GmADF17	0.134747913	1.887491511	0.071389944	154.71
GmADF4/GmADF6	0.006125608	0.049707629	0.123232761	4.07
GmADF4/GmADF10	0.13731522	1.630322318	0.084225812	133.63
GmADF4/GmADF11	0.061047035	0.375395661	0.162620512	30.77
GmADF4/GmADF17	0.13731522	2.03550195	0.067460127	166.84
GmADF6/GmADF10	0.1409996	1.532623422	0.091998855	125.62
GmADF6/GmADF11	0.067713128	0.43668897	0.155060312	35.79
GmADF6/GmADF17	0.1409996	1.874890958	0.07520416	153.68
GmADF10/GmADF11	0.138354154	1.768686102	0.078224256	144.97
GmADF10/GmADF17	0	0.036006914	0	2.95
GmADF11/GmADF17	0.138354154	2.283391828	0.060591508	187.16
GmADF3/GmADF5	0	0.043099828	0	3.53
GmADF3/GmADF12	0.050971746	0.405575985	0.125677427	33.24
GmADF3/GmADF13	0.055782025	0.602577182	0.092572414	49.39
GmADF5/GmADF12	0.050971746	0.442966905	0.115068971	36.31
GmADF5/GmADF13	0.055782025	0.579228993	0.096303923	47.48
GmADF12/GmADF13	0.00298063	0.209949305	0.014196903	17.21
GmADF2/GmADF9	0.100463428	1.282410522	0.078339523	105.12
GmADF2/GmADF16	0.011849173	0.134060542	0.088386728	10.99
GmADF2/GmADF18	0.099344442	1.144883602	0.086772526	93.84
GmADF9/GmADF16	0.105094245	1.249909763	0.084081466	102.45
GmADF9/GmADF18	0.00877203	0.13076504	0.067082378	10.72
GmADF16/GmADF18	0.102841357	1.046334382	0.098287276	85.77

TABLE S3 The similarities among the sequences of the GmADF proteins

Name	*GmADF7*	*GmADF15*	*GmADF8*	*GmADF14*	*GmADF10*	*GmADF17*	*GmADF1*	*GmADF11*	*GmADF4*	*GmADF6*	*GmADF3*	*GmADF5*	*GmADF12*	*GmADF13*	*GmADF2*	*GmADF16*	*GmADF9*	*GmADF18*
GmADF7	100																	
GmADF15	99.28	100																
GmADF8	86.33	87.05	100															
GmADF14	89.93	90.65	96.4	100														
GmADF10	71.74	72.46	76.81	77.54	100													
GmADF17	71.74	72.46	76.81	77.54	100	100												
GmADF1	73.91	74.64	74.64	77.54	78.83	78.83	100											
GmADF11	74.64	75.36	75.36	78.99	79.56	79.56	96.35	100										
GmADF4	76.09	76.09	76.09	77.54	80.29	80.29	88.97	89.71	100									
GmADF6	75.36	75.36	75.36	76.81	79.56	79.56	88.24	88.97	98.54	100								
GmADF3	59.26	59.26	62.96	61.48	59.85	59.85	56.06	56.06	55.3	55.38	100							
GmADF5	59.26	59.26	62.96	61.48	59.85	59.85	56.06	56.06	55.3	55.38	100	100						
GmADF12	58.52	58.52	63.7	62.22	59.26	59.26	57.04	57.78	56.3	56.39	90.91	90.91	100					
GmADF13	57.78	57.78	62.96	61.48	59.26	59.26	57.04	57.78	56.3	56.39	90.21	90.21	99.3	100				
GmADF2	58.39	58.39	64.23	64.23	61.03	61.03	59.56	60.29	60.29	60.29	67.41	67.41	71.11	70.37	100			
GmADF16	59.12	59.12	64.23	63.5	60.29	60.29	58.82	59.56	61.76	61.76	65.19	65.19	69.63	68.89	97.26	100		
GmADF9	58.7	58.7	63.04	63.04	59.12	59.12	56.93	57.66	57.66	57.66	59.18	59.18	61.22	60.54	81.76	80.41	100	
GmADF18	60.14	60.14	64.49	64.49	59.12	59.12	56.93	57.66	57.66	57.66	60.54	60.54	62.59	61.49	82.43	81.08	97.97	100

TABLE S4 RPKM value of *GmADF* genes in nine soybean tissues/organs

Name	roots	root hairs	nodules	stems	leaves	SAM	flowers	pods	seeds
GmADF7	492.36	363.39	215.62	429.25	206.98	224.37	240.43	281.4	185.18
GmADF15	458.82	321.54	238.6	444.16	200.81	225.7	295	243.45	165
GmADF8	204.43	118.44	92.18	187.86	78.64	93.16	107.68	132.59	59.86
GmADF14	50.22	57.97	42.32	125.59	31.53	47.07	50.17	90.21	35.4
GmADF10	0.07	0.00	0.00	0.00	0.00	0.25	9.16	0.00	0.00
GmADF17	0.13	0.00	0.00	0.00	0.00	0.09	7.02	0.02	0.03
GmADF1	0.00	0.00	0.00	0.00	0.00	0.00	21.59	0.01	0.00
GmADF11	0.28	0.06	0.11	0.11	0.37	0.1	50.37	0.19	0.19
GmADF4	0.04	0.00	0.13	0.11	0.23	0.15	8.79	0.11	0.08
GmADF6	0.04	0.02	0.00	0.19	0.00	0.05	49.41	0.03	0.06
GmADF3	18.49	22.40	13.71	20.65	0.89	17.35	9.05	14.23	12.55
GmADF5	23.37	24.99	15.21	25.24	0.91	22.18	10.75	19.73	15.88
GmADF12	17.15	16.52	11.42	60.14	2.82	7.47	10.3	18.72	27.00
GmADF13	14.72	22.79	23.19	32.62	5.11	2.13	31.35	33.76	75.32
GmADF2	69.74	94.25	43.03	79.68	14.64	6.16	26.22	49.97	45.79
GmADF16	19.36	24.88	22.24	49.5	7.94	5.7	26.23	75.56	52.02
GmADF9	81.01	104.12	122.86	126.03	97.82	70.45	110.88	80.68	250.9
GmADF18	40.03	71.19	80.27	81.14	34.13	51.03	65.73	51.73	165.78
